# A Lucky Accident: Brugada Syndrome Associated with Out-of-Hospital Cardiac Arrest

**DOI:** 10.1155/2018/1465867

**Published:** 2018-09-19

**Authors:** Michelle T. Lee, Naddi Marah

**Affiliations:** ^1^Department of Internal Medicine, University of Texas Health Science Center at the Heart and Vascular Institute, Houston, Texas, USA; ^2^Division of Cardiovascular Medicine, University of Texas Health Science Center at the Heart and Vascular Institute, Houston, Texas, USA

## Abstract

About 350,000 cases of out-of-hospital cardiac arrest (OHCA) occur yearly in the United States. Unfortunately, even with treatment from emergency medical service (EMS) staff and hospitalization, only 12% survive past discharge for multiple reasons. Classically, Brugada syndrome (BrS) initially presents as a new syncopal episode in young males without obstructive coronary artery disease (CAD). However, in this case report, a patient who emergently presented with a ST-elevation myocardial infarction (STEMI) challenges the stereotypical presentation. Despite successful stent placement for relatively minor obstructive CAD, new ST-segment elevations appeared on electrocardiogram (ECG) and persistent ventricular fibrillation arrests may signify an additional underlying pathology of BrS.

## 1. Presentation

A 54-year-old man with no prior past medical history presented to the emergency department after suffering from an OHCA. As the driver of a single-vehicle accident, he lost control after suddenly slumping over the wheel. Fortunately, a police officer nearby initiated CPR with return of spontaneous circulation (ROSC) achieved shortly afterwards. When EMS arrived, he was unresponsive, hypertensive (170/90 mmHg), and tachycardic (140 beats per minute, sinus rhythm). Emergent intubation in the field was required due to failure of airway protection. Afterwards, an air ambulance transported the patient to our hospital for immediate cardiac catherization.

## 2. Assessment

Less than 90 minutes elapsed from time of initial collapse to percutaneous coronary intervention (PCI). Coronary angiography revealed 70% stenosis in the proximal right coronary artery (RCA) without obstruction elsewhere. A drug-eluting stent (DES) was successfully placed with resultant 0% stenosis in the RCA and TIMI 3 flow. Additionally, telemetry monitoring showed complete resolution of ST-segment changes to normal sinus rhythm. Dual-antiplatelet therapy load of aspirin 325 mg and ticagrelor 180 mg were also given prior to moving the patient to PACU, where he recovered without complications. Since the patient suffered an OHCA with uncertain time to ROSC, therapeutic mild hypothermia (TMH) protocol was initiated upon his arrival to the CCU.

Several hours after TMH protocol initiation, a code blue was called due to ventricular fibrillation (VF) arrest. Ten minutes elapsed until ROSC to sinus tachycardia (170 beats per minute); he received four rounds of CPR, three defibrillator-delivered electrical shocks (300 J), and two epinephrine injections. Stat labs revealed no electrolyte derangements. Several minutes later, the rhythm degenerated into VF once more. ROSC to normal sinus rhythm (NSR) occurred shortly afterwards with one round of CPR and one AED-administered electrical shock at 300 J.

**Figure 1:**
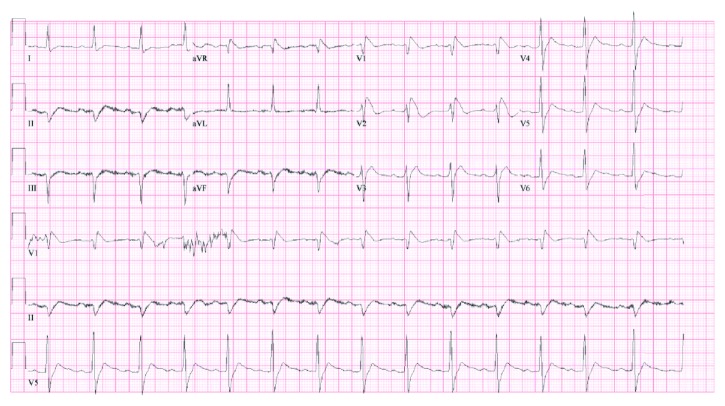
ECG during in-hospital VF arrest: new-onset coved-type ST-segment elevations at anterior precordial leads.

Shortly after ROSC, repeat labs, CXR, and transthoracic echocardiography were obtained and unremarkable; all cardiac chambers were normal in function and size. In particular, LVEF was 60–65% without wall motion abnormalities and no signs of RVOT abnormalities were noted. Head CT was negative for hypoxic ischemic changes or other pathology. However, an ECG obtained during the first code blue showed ST-segment elevations with a curiously peaked downsloping shape in the anterolateral chest leads (Figure 1). Besides indicating a possible right fascicular blockade, this pattern suggested a critical underlying pathology.

## 3. Diagnosis

Patients diagnosed with BrS must satisfy both of the following criteria: (1) typical ECG findings of BrS and (2) clinical features at time of diagnosis. Often, this entails a spontaneous episode of syncope, VF, or polymorphic ventricular tachycardia (VT) preceding ECG findings suggestive of BrS. Alternatively, asymptomatic high-risk patients may undergo a monitored sodium channel blocker challenge; if aforementioned events occur during the test, an inferred diagnosis of BrS is made [1–3].

Two types of Brugada ECG patterns exist: Type 1 consists of the hallmark “coved-type” ST-segment elevations with negative T-wave in at least one right precordial lead (V1-V2). Type 2 BrS has a “saddle back” ST-T-wave configuration with either an upright or biphasic T-wave.

At least one of the following clinical requirements must be met: (a) history of VT/VF, (b) family history of SCD, (c) family history of coved-type ECG, (d) agonal respiration during sleep, or (5) inducibility of VT/VF during electrophysiological study. Those with BrS classically have normal cardiac structure and function on TTE and lack ischemic cardiomyopathy [1].

In this patient's case, his history and clinical findings are most consistent with Type 1 BrS. The classic coved-type ST-segment elevations are evident on ECG and there was positive personal history of VF arrest [2, 4]. Further investigation by our electrophysiologists confirmed the BrS diagnosis.

## 4. Management

**Figure 2:**
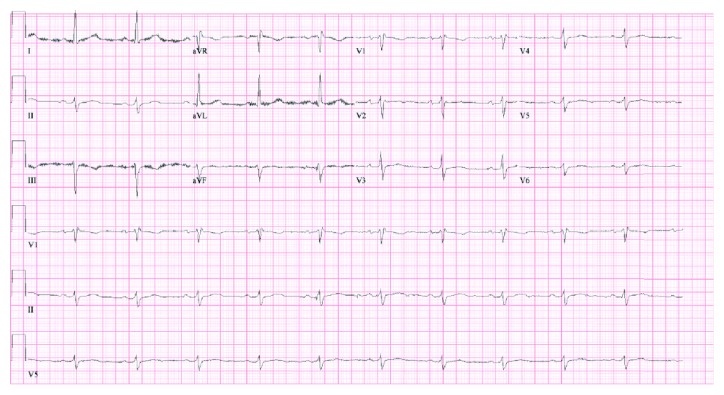
Resolution of BrS Type I ECG pattern.

Patients diagnosed with symptomatic BrS require an implantable cardioverter defibrillator (ICD) as first-line management (Class I recommendation) [4, 5], which our patient received prior to discharge. Around the time of ICD placement and one week after initial presentation, the ECG findings suggestive of BrS disappeared without further intervention (Figure 2).

Patients who experience appropriate recurrent ICD shocks may need additional pharmacological rhythm control, usually quinidine or amiodarone. Low-risk patients with only positive findings but lack symptoms or strong family history of sudden cardiac death should be monitored with regularly scheduled follow-up appointments without specific therapy. Patients without symptomatic BrS but have significant risk factors, such as positive family history, usually undergo further risk stratification with an invasive electrophysiological study [2, 4–6].

## 5. Discussion

In 1992, Pedro and Josep Brugada first described this autosomal dominant arrhythmia.

in a series of eight patients who suffered from VF arrest. All ECGs from those patients possessed a characteristic right bundle-branch block with coved-type ST-segment elevation on ECG [7]. Years later, discovery of the *SCN5A* gene in 1998 provided further insight into this syndrome and association with sudden cardiac death (SCD). By encoding the fast-type sodium channel's *α*-subunit, mutations in *SCN5A* led to slow conduction, particularly in the right ventricular outflow tract (RVOT). To date, although over 10 genes have since been linked to BrS, *SCN5A* remains the most commonly associated gene. Ultimately, those with BrS usually suffer from syncopal episodes, palpitations, or SCD attributed to VF. Young adult men, who are nine times more likely than females to be affected, usually present after a syncopal episode. Coronary angiography, if performed, usually shows nonobstructive CAD. Additionally, family history is often significant for early cardiac death or early death without known cause [1–3, 6, 8].

In this case, the patient presented later in life status post OHCA with minimal 1-vessel CAD and lacked familial cardiac disease history, unlike most textbook presentations. Moreover, despite a routine successful PCI, he suffered multiple subsequent VF arrests with return of ST-segment elevations with morphology consistent with Type I BrS.

Notably, Brugada-type ECG changes can appear in various conditions, including fever, stress, certain medications, and acute ischemic lesions of the RVOT. Although the latter scenario may induce ST-elevations in right precordial leads, mimicking BrS, subsequent ventricular tachycardia (VT) or VF episodes occur in a subset of patients without obstructive CAD and/or with diagnosed BrS. Post-RVOT acute ischemia complicated by VT/VF has not been documented in cases with obstructive CAD [9]. Since this patient only had a marginal obstructive lesion of the RCA, the etiology of his initial cardiac arrest and subsequent VF episodes with coved-type ST-segment elevations is highly suspicious for BrS.

## 6. Conclusion

BrS is a common yet preventable cause of SCD; hence, it is important to recognize its presentation and characteristic diagnostic findings. Though classically associated with patients presenting post OHCA without obstructive CAD, BrS may also initially present in a patient with mild CAD complicated by new-onset VT/VF and Brugada-pattern ECG post intervention. Such situations warrant further investigation with BrS in the differential.
