# Erratum to “A Lucky Accident: Brugada Syndrome Associated with Out-of-Hospital Cardiac Arrest”

**DOI:** 10.1155/2018/9837840

**Published:** 2018-12-18

**Authors:** Michelle T. Lee, Naddi Marah

**Affiliations:** ^1^Department of Internal Medicine, University of Texas Health Science Center at the Heart and Vascular Institute, Houston, Texas, USA; ^2^Division of Cardiovascular Medicine, University of Texas Health Science Center at the Heart and Vascular Institute, Houston, Texas, USA

In the article titled “A Lucky Accident: Brugada Syndrome Associated with Out-of-Hospital Cardiac Arrest” [1], there was an error in the first paragraph in the Discussion, where its first sentence was split. This was a production error. Therefore, the first sentence of the first paragraph in the Discussion should be corrected as follows:

“In 1992, Pedro and Josep Brugada first described this autosomal dominant arrhythmia in a series of eight patients who suffered from VF arrest.”

## References

[1] Lee M. T., Marah N. (2018). A lucky accident: Brugada syndrome associated with out-of-hospital cardiac arrest. *Case Reports in Cardiology*.

